# Indira’s story

**Published:** 2017-04-20

**Authors:** Russell Dawe

**Affiliations:** 1 Memorial University of Newfoundland, Newfoundland and Labrador, Canada

## Abstract

Indira is an independent woman who does not live a traditional Nepali life. She rescues abandoned and abused young women from sexual exploitation and provides them with love, support, and education. Her story highlights the key role of the social determinants of health in caring for marginalized populations. Challenges and benefits of attempting to learn from another’s personal narrative are also considered.

Family doctors practice at the crossroads of medicine and the social determinants of health. Our focus is not on an illness but on a person. We need to hear the stories of our patients’ lives so that we can provide patient-centered care. We care about issues such as empowerment and education, compassion and a safe place for our patients to call home. We are advocates – a challenging role for most physicians.^1^ As a medical educator, I must find ways of teaching advocacy and, hopefully, motivating learners to put it into practice, wherever they may be.

In April 2016, I took my family to Nepal to establish some working relationships and, hopefully, gain some perspective for my teaching. My most memorable encounter on this trip was with Indira Ghale, an independent woman who does not live a traditional Nepali life. A colleague told me about this woman, who rescues girls from abusive situations in rural villages and from human trafficking in Kathmandu, so I gratefully accepted a dinner invitation and soon we were warmly welcomed into her home.

Indira says it all began with her own mother, “When I look back on my mother’s life, she got married at the age of fifteen, and she had no opportunity to say, ‘No, I don’t want to get married to a man who’s already got women.’” Indira’s mother married and had her first child at sixteen. Because her husband wanted a son, she went on to give birth to eight more children without resting. But Indira’s mother had a vision for something better for her daughters.

“My mother started thinking and educated us. And every time she said, ‘You have to go to school. You have to study hard…. You can get opportunity…. You will have choice.’”

Indira has followed in her mother’s footsteps, becoming a woman of strength who invests in the education of young women in need. She shares her home with seven abandoned and abused young women and offers them love, support, and education. She has lived through discrimination in many forms, but she doesn’t view herself or her daughters as weak. “I still have some kind of energy, power,” Indira says. “That power comes from my education.”

As Indira talks, my own daughters interrupt, raising their voices to complain that they’re hungry. “Hush, we’ll eat soon. Don’t cause a fuss.”

“No,” Indira says. “I want my girls to see. Your children speak so frankly…. Mine must learn to speak for themselves too. In our society we have very less opportunity to say ‘no.’” There is much discrimination throughout the world. In Nepal, gender, caste, and wealth allow some to take advantage; society’s blind eye can be a cruel response of wilful ignorance. In Kathmandu, Indira has friends from women’s rights organizations who see the invisible and reach out to the untouchable; those deemed worthless and discarded by society.

The joy and beauty of Nepal and its people are evident in the faces of our hosts. However, Indira goes on to explain that these young girls have also faced many challenges in their rural villages or upon moving to Kathmandu. Because these girls are under the age of sixteen, and without paperwork, they easily gain employment in restaurants. The restaurant owners are waiting to take advantage of their vulnerability. “And they cannot [say], ‘No I don’t want to do, I don’t want to sit together with the customer and be together [so that] the customer can do whatever he wants to do [and] touch my body.’ …They have to have sex with the customer, and the benefit always goes to the owner. …For some time they resists, but what the owner do is they give alcohol and also the drug to make them [cooperate].” This was the very life two of Indira’s daughters were rescued from.

I watch the older girls as they experiment with different toys to see which will hold the interest of my three year-old. They laugh, so happy now. I would never have guessed what they’ve suffered. But now I can see the strength in their smiles.

Indira goes on to relate the stories of her other three daughters, who came to her when they were just seven, nine and ten years old. Two girls were raped by their grandfather, and one by her brother. These girls were then rejected, not only by their family members, but also from Nepalese society. “They don’t want to see the girl’s face within their community…. The society blamed them, ‘it’s your fault’. Never blame to the man.”

I look at my own daughters, so innocent, playing with these girls. Sometimes I am shocked by the world we live in. I want to raise my own children to understand these things, and yet I want to protect them too.

Indira tells me that the girls are beginning to win back societal acceptance and recognize for themselves that they are not at fault for the abuse they’ve endured.

“They are very strong daughters,” she tells me.

Three of the rapists are now in jail because these girls had the courage to speak up. One remains free because of his wealth and connections, but the girl he raped is not giving up. She tells Indira, “I want to be lawyer and I will provide the justice for girls who are suffering like me.”

Empowerment and education. Compassion and a safe place to call home.

Indira’s story tells of a woman who was willing to hear the pain of others and respond in her life, with a story of her own. Hearing, however, requires listening. How do we do that? Our own cultural assumptions filter another’s life in a way that makes sense to us, and always there is the risk that we misunderstand or respond in well-intentioned but harmful ways. Even as I tell the story, why have I focused on some parts of Indira’s story more than others? The fact that I am making such decisions reflects a power imbalance that I find distasteful. However, even as Indira reviews this manuscript for her approval, I see my fingerprints on her story like smudges on a window, partially obstructing the reader’s view. Perhaps by acknowledging those smudges I can write, with a little more integrity, not only her story, but also our story: where two stories intersect and a new relationship, a new narrative begins.

The social determinants of health, including the issues of education, gender, finance, and ethnicity seen in Indira’s story, represent power. We may lack an objective metric to assess the inequity here, but when we hear a story we begin to see, albeit through our own tainted lens, the complex ways health is impacted. However, knowledge – even of a complex concept – is not enough. Medical educators must help learners make the leap from learning (knowledge) to application (behaviour). It is relatively easy to state things we accept as correct. E.g., living in a rural village can make education difficult; financial need can lead to abusive employment circumstances; and the victims of abuse are often blamed or even shunned. Propositional truths like these are useful at times, but as Andy Goodman once said, “nobody ever marched on Washington because of a pie chart.”^2^ If our lives are not impacted by the stories we hear, then perhaps we would be better off ignorant rather than steeling ourselves against compassion. Inaction leaves us voyeurs, consuming stories for interest but not shaping our own lives in response.^3^ However, when we let the lives of others influence how we live, we can follow Indira’s example and become advocates. Indira demonstrates great courage in the face of tremendous adversity, working outside of social norms to break down gender barriers and effect social change. As a medical educator, if narratives like this will help me teach advocacy, I consider the above risks, although real, worth taking.

Indira tells the story of one woman’s struggle for education and a home. Once she obtained these, she did not keep them to herself but was moved by the stories of those around her to join them in their struggle. Her own relative power was not wasted on inaction, but compelled her to reach out. Empowerment and education. Compassion and a safe place to call home. I suspect Indira’s commitment to meeting these needs has done more for the health of her daughters than any doctor’s prescription.
